# mTOR Inhibition Attenuates Dextran Sulfate Sodium-Induced Colitis by Suppressing T Cell Proliferation and Balancing TH1/TH17/Treg Profile

**DOI:** 10.1371/journal.pone.0154564

**Published:** 2016-04-29

**Authors:** Shurong Hu, Mengmeng Chen, Yilin Wang, Zhengting Wang, Yaofei Pei, Rong Fan, Xiqiang Liu, Lei Wang, Jie Zhou, Sichang Zheng, Tianyu Zhang, Yun Lin, Maochen Zhang, Ran Tao, Jie Zhong

**Affiliations:** 1 Department of Gastroenterology, Ruijin hospital, Shanghai Jiaotong University School of Medicine, Shanghai, PR China; 2 Department of Surgery, Cancer hospital, Fudan University, Shanghai, PR China; 3 Department of Surgery, Ruijin hospital, Shanghai Jiaotong University School of Medicine, Shanghai, PR China; 4 Department of Hepatobiliary-Pancreatic Surgery, Zhejiang Provincial People’s Hospital, Hangzhou, Zhejiang Province, PR China; Wayne State University, UNITED STATES

## Abstract

It has been established that mammalian target of Rapamycin (mTOR) inhibitors have anti-inflammatory effects in models of experimental colitis. However, the underlying mechanism is largely unknown. In this research, we investigate the anti-inflammatory effects of AZD8055, a potent mTOR inhibitor, on T cell response in dextran sulfate sodium (DSS)-induced colitis in mice, a commonly used animal model of inflammatory bowel diseases (IBD). Severity of colitis is evaluated by changing of body weight, bloody stool, fecal consistency, histology evaluation and cytokine expression. We find that AZD8055 treatment attenuates DSS-induced body weight loss, colon length shortening and pathological damage of the colon. And AZD8055 treatment decreases colonic expression of genes encoding the pro-inflammatory cytokines interferon-γ, interleukin (IL)-17A, IL-1β,IL-6 and tumor necrosis factor(TNF)-a and increases colonic expression of anti-inflammatory cytokines IL-10. We show that AZD8055 treatment decreases the percentages of CD4+ T cells and CD8+ T cells in spleen, lymph nodes and peripheral blood of mice. We also find that AZD8055 treatment significantly reduces the number of T helper 1(TH1) cells and TH17 cells and increases regulatory T (Treg) cells in the lamina propria and mesenteric lymph nodes. Furthermore, we demonstrates that AZD8055 suppresses the proliferation of CD4+ and CD8+ T cells and the differentiation of TH1/TH17 cells and expands Treg cells in vitro. The results suggest that, in experimental colitis, AZD8055 exerts anti-inflammatory effect by regulating T helper cell polarization and proliferation.

## Introduction

Inflammatory bowel diseases (IBD) which consist of Crohn’s disease (CD) and ulcerative colitis (UC), are chronic heterogeneous intestinal disorders, which remain clinically challenging [1, 2]. Currently, the drugs for IBD patients are limited. The precise etiology of IBD remains unknown, although it is generally accepted that it result from an overactive immune response to commensal bacteria within the gut in genetically predisposed individuals [3].

Helper T cells have a significant role in IBD pathogenesis [4]. TH1, TH2, TH17 and regulatory T cells (Tregs) form an important quarter of helper T cells [5, 6]. Studies have been shown that TH1, TH2, and TH17 cells were essential for defenses against excessive entry of microorganisms [7, 8]. Intestinal immune homeostasis depends on the regulation and balance of these T cell subgroups. It has been shown that deregulated overexpansion and activation of effector cells in relation to regulatory T cells can lead to intestinal inflammation like IBD [9, 10].

The T cell transfer induced colitis has been used to study T cell response in IBD. In this study, CD4+CD45RBhi T cells are transferred into immune-deficient mice. Since this model depends on genetically compromised mice and an unbalance of naïve and Treg cells which is not seen in wild type mice, it does not reflect the immunological courses of the development of pathogenic T cells in healthy animals [11–13]. On the other hand, DSS-induced colitis model is a classic and stable model of murine colitis, which can be used in all backgrounds of mice. Many drugs used in IBD patients are also available for this model [14–16]. Previous studies have shown that DSS-induced colitis is often not considered as a good model for T cell involvement, since it is chemical damage model which can be induced without the help of T cells. However, recent studies show that T cells especially pro-inflammatory, antigen-specific CD4+ T cells accumulate at the site of inflammation, and do progress during DSS-induced colitis model, suggesting that DSS model can be used to study T cell development during intestinal inflammation [17–19].

Mammalian target of Rapamycin (mTOR) is a protein kinase that regulates cell survival, cell growth, cell proliferation and autophagy. Besides its crucial role in tumorigenesis, recent studies show that mTOR participates in adjusting adaptive immune response and modulating CD4+ or CD8+ T cell polarization, as well as increasing the percentage of Treg cells [20–22]. Farkas et al showed that Rapamycin, an mTOR inhibitor, reduced leukocyte migration as effectively as immunosuppressant cyclosporine A (CsA) in DSS-induced murine colitis [23]. Matsuda et al found that Everolimus, a Rapamycin analog, prevented colitis in interleukin-10(IL-10)–/–model by decreasing the percentage of CD4+ T cells in the colonic mucosa and reducing IFN-γ production [24].

mTOR functions in two multi-protein complexes, mTORC1 and mTORC2. mTORC1 is suppressed by Rapamycin, but Rapamycin can’t block mTOR activity completely due to its inability to influence mTORC2 directly [25,26]. On the other hand, ATP-competitive mTOR inhibitor AZD8055 targets the ATP site and inhibits any mTOR-containing complex [27]. AZD8055 not only inhibits phosphorylation of the mTORC1 substrates p70S6K and 4E-BP1, but also phosphorylation of the mTORC2 substrates AKT and downstream protein [28]. In spite of the emerging role of RAPA-resistant mTOR in immune cell function, the effect of AZD8055 on T cells has not been fully studied. In this study, we investigate the effect of AZD8055 in DSS-induced colitis. We find that AZD8055 attenuates DSS-induced colitis by inhibiting T-cell proliferation and balancing TH1/TH17/Treg profile.

## Materials and Methods

### Ethics Statement

All experimental procedures were performed in accordance with the criteria issued by the Chinese ethics committee for animal studies, formulated by the Ministry of Science and Technology of the People’s Republic of China. The animal protocols were examined and approved by the Ethical Committee on Animal Experiments at Shanghai Ruijin Hospital. All endeavors were made to alleviative suffering.

### Mice

6–8 weeks old male C57BL/6 mice were gained from Shanghai Laboratory Animal Center (SLAC) and housed under specific pathogen-free environment in the Research Center for Experimental Medicine of Shanghai Ruijin Hospital. All animal studies were authorized and handled according to the guidelines of the Ethics Committee of Ruijin Hospital.

### Reagents

AZD8055 was purchased from Selleck (China). It was dissolved in dimethyl sulfoxide (DMSO) and prepared as 10mM stock solutions and stored at -20°C for in vitro studies. For in vivo administration, AZD8055 solution was diluted in sterile emulsifiers.

### Induction of Dextran Sodium Sulfate (DSS) Colitis and assessment of acute colitis

Mice were randomly divided into three groups, Wild type (WT), DSS, DSS+AZD8055 (n = 8 in each group). For acute colitis, mice were subjected to 4% (w/v) DSS (MW 36, 000–50, 000, MP Biomedical) in their drinking water for 7 (day0–6) days. Mice were monitored daily by change of body weight, gross rectal bleeding, and stool consistency. The disease activity index score (DAI) was described previously (Table 1) [29]. Mice were treated daily intra-peritoneally (i.p.) with 10mg/kg AZD8055 or emulsifier as control from day 0 to day 6 and were sacrificed on day 7.

**Table 1:** The disease activity index score (DAI).

Weight loss(%)	Occult blood	Stool consistency	Score
<1	Negative	Normal	0
1–5	Haemoccult positive	Soft stool	1
5–10		Loose stool	2
10–20		Muddy stool	3
>20	Gross bleeding	Diarrhea	4

Five grades of weight loss and stool consistency and three grades of occult blood.

### Histological examination

The colons were embedded in paraffin and stained with hematoxylin-eosin (H&E). To evaluate the histological inflammation, we adopted the histological damage score: edema; crypt loss; erosion/ulceration and mono- and poly-morphonuclear cells infiltration. The degree of colon inflammation scored from 0 to 20 [30]. The analysis was performed by two investigators who were blinded to the experiment.

### Preparation of lamina propria (LP) lymphocytes

LP lymphocytes were isolated according to previously described study [31]. Briefly, the intestines were opened longitudinally, washed with PBS to remove stool, and shaken in Hank's Balanced Salt Solution (HBSS) supplemented with 4% FBS and 5mM EDTA (Sigma-Aldrich) at 37°C for 25 min. This procedure was to remove epithelial cells and intraepithelial lymphocytes. The colon fragments were then incubated with RPMI 1640 supplemented with 4% FBS and 1mg/ml collagenase type IV (R&D) and Dnase I (Sigma-Aldrich) at 37°C for 25 min with stirring. The digested tissues were pooled together and separated on a 40/80% discontinuous Percoll gradient (GE Healthcare). The gradient separation was centrifuged at 2500rpm for 25 min at room temperature. Lamina propria mononuclear cells were collected at the interface of the Percoll gradient, washed, and suspended in RPMI 1640 containing 4% FBS.

### Establishment of T cell Proliferation and Differentiation in vitro

Splenocytes and lymph nodes were isolated and prepared as mononuclear cells from mice. CD4^+^ T cells and CD8^+^ T cells were respectively negatively selected using the CD4^+^ T cell Isolation kit (Miltenyi) and CD8^+^ T cell Isolation kit (Miltenyi). In order to analysis T cell proliferation, CD4^+^ T cells and CD8^+^ T cells were labeled with carboxyfluorescein succinimidyl ester (CFSE; Invitrogen). Cells were stimulated with mouse antibody CD3/CD28 (Invitrogen) at 2ug/ml for 3 days in the presence of AZD8055 at the indicated concentrations and were evaluated by flow cytometry. In order to analysis T cell differentiation into TH1, TH17 or Treg cells, naive T cells were sorted by CD4^+^CD62L^+^T cell Isolation kit II (Miltenyi). Naive T cells were labeled with CFSE and stimulated with anti-CD3 (2 μg/mL), and anti-CD28 (2 μg/mL) antibodies under TH0(40 U/mL mouse IL-2), TH1 (40μg/mL anti-mouse IL-4, 40 ng/mL mouse IL-12), TH17 (50 ng/mL mouse IL-6, 2.5 ng/mL mouse TGF-β, 10 μg/mL anti-mouse IFN-γ, 20 ng/mL mouse IL-23, 10μg/mL anti-mouse IL-4) or Treg conditions(2 ng/mL mouse TGF-β) for 4 days in the presence of AZD8055 at specified concentrations and were evaluated by flow cytometry.

### Flow cytometry

To quantify the percentage of CD4 and CD8 positive cells, mononuclear cells from spleen, mesenteric lymph node and lamina propria were washed and incubated with anti-CD3-APC, anti-CD4-FITC, anti-CD8-PE (ebioscience, San Diego, CA). To determine the percentage of TH1 and TH17 cells, mononuclear cells were stimulated with PMA (50ng/mL, sigma) and Ionomycin (1μg/mL, Tocris) and BFA (1:1000, eBioscience) for 6 hours. Cells were then washed and stained with anti-CD4-FITC. Following CD4 staining, cells were blocked, fixed, permeabilized and stained with anti-IFN-γ-APC or anti-IL-17-PE-Cy7. To detect the percentage of Treg cells, mononuclear cells were stained with anti-CD4-FITC and anti-CD25-APC and then fixed/permeabilized and incubated with anti-Foxp3-PE. All labeled cells were detected using FCM on the FACScan Flow Analyzer. The results were evaluated with FlowJo7.6 software [32, 33].

### Quantitative reverse-transcription polymerase chain reaction analysis

Total RNA from colonic samples were extracted by TRIzol^®^ Reagent (Invitrogen Life Technologies) and were reverse transcribed using oligo (dT) primers (Takara) in accordance with the manufacturer's instructions. Real-time polymerase chain reaction (PCR) was performed on QuantiTect SYBR Green PCR Kit. The expressions of target gene were analyzed by their ratios to the house keeping gene hypoxanthine-guanine phosphoribosyl transferase (HPRT). The sequence of target genes primer sets were listed in Table 2.

**Table 2:** Sequence of primer pairs used in real-time quantitative PCR.

Gene name	Forward primer(5’→3’)	Reverse primer(5’→3’)
TNF-α	CTCTTCAAGGGACAAGGCTG	CTCTTCAAGGGACAAGGCTG
IL-1β	TTCAGGCAGGCAGTATCA	GTCACACACCAGCAGGTTA
IL-6	CCAATGCTCTCCTAACAGA	TGTCCACAAACTGATATGC
IFN-γ	CAGCAACAACATAAGCGTC	CTCAAACTTGGCAATACTC
IL-17A	CCTTCACTTTCAGGGTCGAG	CAGTTTGGGACCCCTTTACA
IL-10	AGGGCACCCAGTCTGAGAACA	CGGCCTTGCTCTTGTTTTCAC
HPRT	TCAACGGGGGACATAAAAGT	TGCATTGTTTTACCAGTGTCAA

### Statistical Analysis

Statistical analysis was determined by t-test and one-way analysis of variance. Differences with P<0.05 were considered statistically significant.

## Results

### 1. AZD8055 attenuates the development of intestinal inflammation

**Fig 1:**
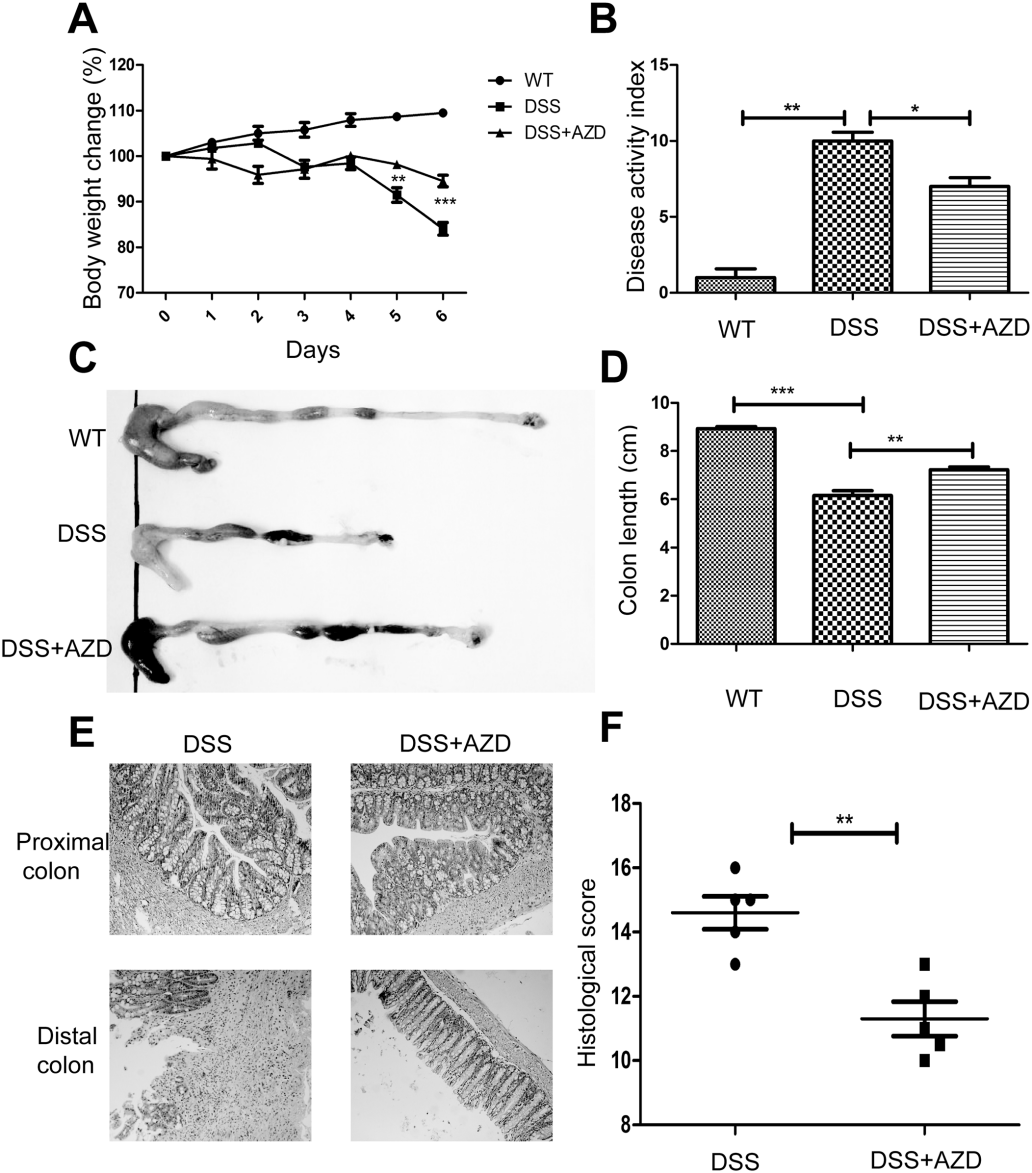
AZD8055 attenuates the development of intestinal inflammation. When the mice developed clinical signs of disease, they were sacrificed. (A) During the course of colitis, body weights were recorded and calculated as percentage of the initial weight at day 1 (n = 8 for AZD, n = 8 for control). (B) Disease activity index (DAI) of each group. (C) Gross morphology of each group. (D) The length of colon of each group. (E) Histological observations of colon sections with H&E staining (Original magnifications, 200×magnification). (F) Histological score of each group. All data represented the mean±SEM. Statistical significance was assessed by t-test. *P<0.05, **P<0.01.

In assessing the role of AZD8055 in the development of colitis, we used 4% DSS to induce colitis. It is known that DSS can induce severe inflammation in mice characterized by persistent weight loss, rectal bleeding and diarrhea. As shown in Fig 1A, DSS-treated mice exhibited profound body weight loss, whereas AZD8055 attenuated the loss of body weight. By daily monitoring of clinical manifestations such as weight loss, diarrhea, and rectal bleeding, we scored disease activity index (DAI) according to the standards in Table 1. We observed that AZD8055 decreased DAI score and prevented colitis (Fig 1B). DSS-induced colon shortening, a marker of intestinal inflammation, was also ameliorated by AZD8055 (Fig 1C and 1D). The severity of intestinal inflammation and ulceration were further assessed by histological study using Haematoxylin & eosin (H&E) staining. DSS-induced pathological damage includes epithelial crypt loss, gross ulceration following massive infiltration of monocytic cells into the mucosa, as well as edema and congestion of the submucosa. As shown in Fig 1E and 1F, AZD8055-treated mice showed less inflammatory cells infiltration and smaller ulceration and greater mucosal integrity. Thus, we concluded that AZD8055 treatment could relieve DSS-induced colitis.

Results were from three independent experiments.

### 2. AZD8055 reduces pro-inflammatory mediator production in colon

**Fig 2:**
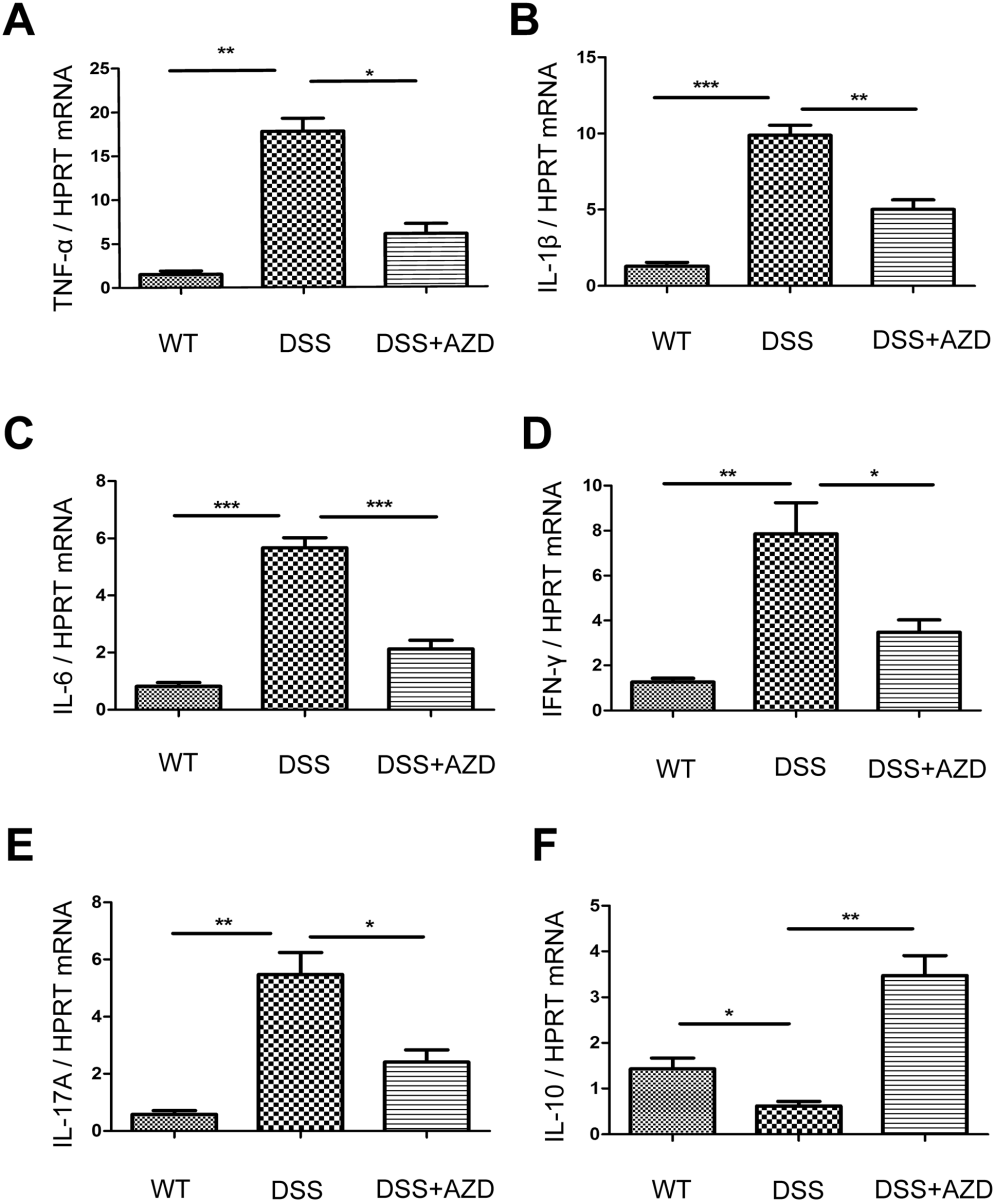
AZD8055 treatment suppresses the expression of pro-inflammatory mediators. **(A-F)** The expression profiles of IFN-γ, TNF-α, IL-1β, IL-6, IL-17A and IL-10 were determined in the colon of emulsifier or AZD8055-treated mice by real-time PCR. Data was normalized to the expression of HPRT mRNA (n = 8/group). All data represented the mean±SEM. Statistical significance was assessed by t-test. *P<0.05, **P<0.01.

The hallmark of DSS-induced colitis is the increased production of pro-inflammatory cytokines in the colon. The colon was isolated and real-time PCR was used to assess the expression profiles of pro-inflammatory cytokines. As shown in Fig 2, the mRNA levels of pro-inflammatory cytokines, such as IFN-γ, TNF-α, IL-1β, IL-17A and IL-6 in the colon increased in the presence of DSS administration, whereas AZD8055 treatment decreased the mRNA levels of these cytokines. And the anti-inflammatory cytokine IL-10 increased under AZD8055 treatment.

### 3. AZD8055 decreases the proportion of CD4^+^ T cells and CD8+ T cells in vivo

**Fig 3:**
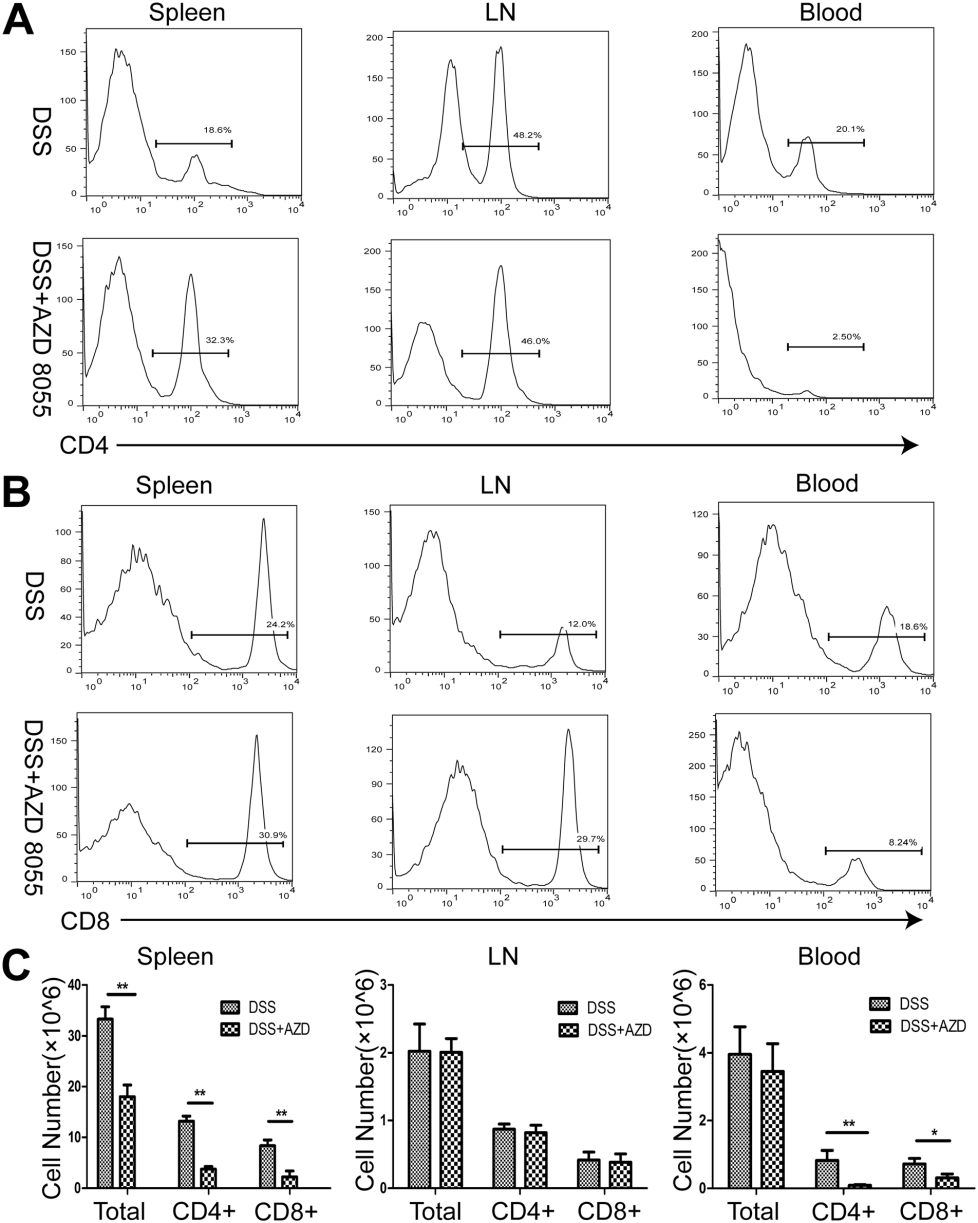
AZD8055 treatment leads to a decrease in the percentage of CD4+ T cells and CD8+ T cells in vivo. (A) The percentage of CD4^+^ T cells in the spleen, lymph nodes and peripheral blood of mice treated with AZD8055 or emulsifier. (B) The percentage of CD8^+^ T cells in the spleen, lymph nodes and peripheral blood of mice treated with AZD8055 or emulsifier. (C) The cell number of CD4+ T cells and CD8+ T cells in the spleen, lymph nodes and peripheral blood of mice treated with AZD8055 or emulsifier. All data represented the mean±SEM. Statistical significance was assessed by t-test. *P<0.05, **P<0.01.

Aberrant immune response is responsible for chronic inflammatory diseases. CD4^+^ and CD8^+^ T cells played important role in inflammatory bowel disease. We isolated and prepared mononuclear cells of spleen, peripheral lymph nodes and peripheral blood from DSS-treated and DSS+AZD8055 treated mice. The results showed that, compared with DSS treatment, DSS+AZD8055 induced reduction in the number and proportion of CD4+ and CD8+ T cells, especially in the spleen and peripheral blood (Fig 3).

### 4. AZD8055 suppresses the proliferation of CD4^+^ T cells and CD8+ T cells in vitro

**Fig 4:**
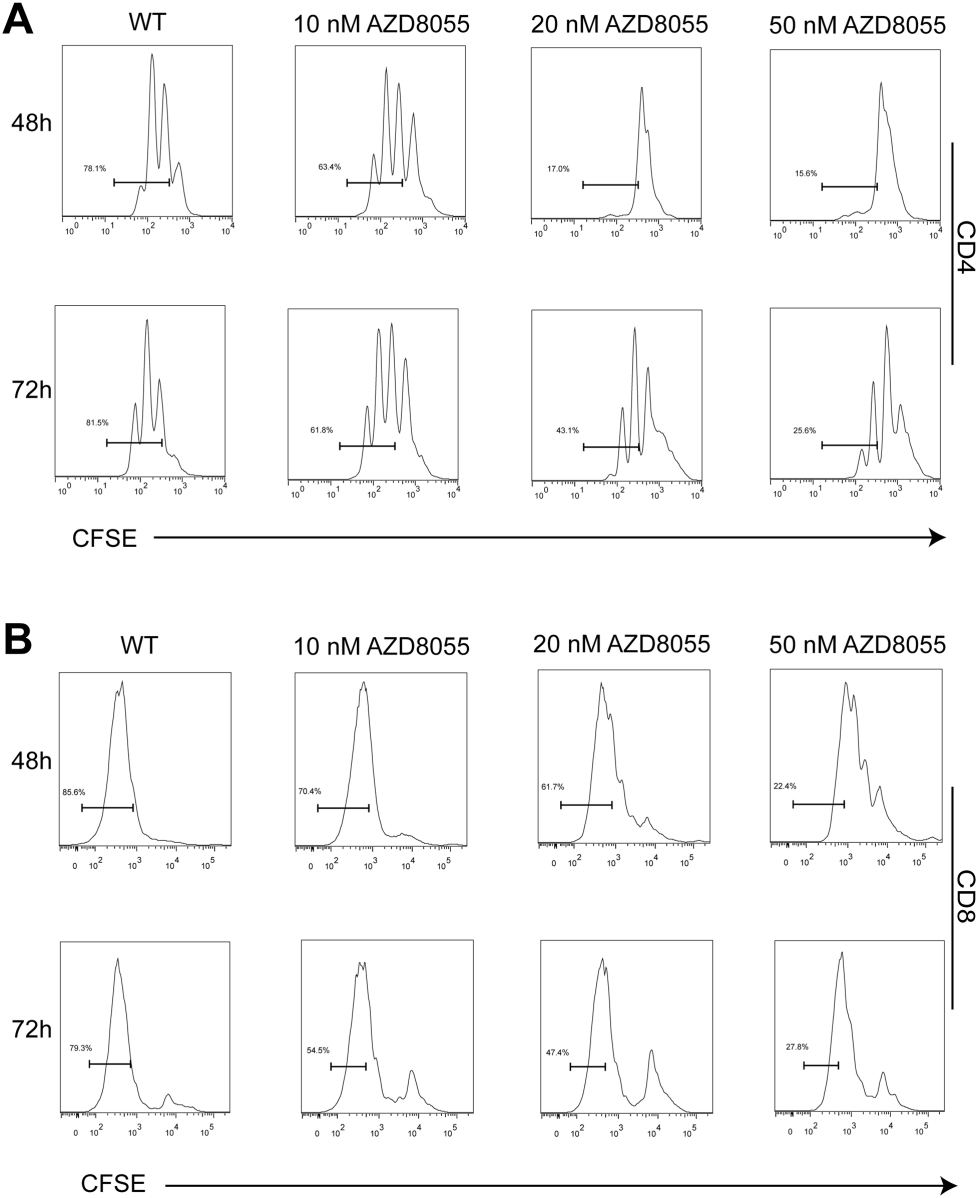
AZD8055 inhibits the proliferation of CD4^+^ T cells and CD8^+^ T cells in vitro. CD4^+^ T cells and CD8^+^ T cells isolated from spleen and mesenteric lymph node of mice were stimulated with anti-CD3/CD28 and labeled with CFSE in the presence of AZD8055 (10–50nM) for two or three days. (A) The proliferation of CD4^+^ T cells in the presence of AZD8055 were examined by flow cytometry. (B) The proliferation of CD8^+^ T cells in the presence of AZD8055 were examined by flow cytometry. Results were from three independent experiments.

As described above, AZD8055 decreased the percentage of CD4^+^ and CD8^+^ T cells in spleen, lymph nodes and peripheral blood. To further confirm the anti-inflammatory effect of AZD8055 in vitro, we investigated if AZD8055 inhibited the proliferation of CD4^+^ and CD8^+^ T cells. CFSE-labeled CD4^+^ and CD8^+^ T cells were activated with anti-CD3/CD28 beads in the presence or absence of increasing concentrations of AZD8055 (10–50nM). The proliferation of CD4^+^and CD8+ T cells were analyzed by flow cytometry. As shown in Fig 4A and 4B, we saw that the proliferation of CD4^+^ and CD8^+^ T cells were inhibited by AZD8055 in a dose-dependent manner.

### 5. AZD8055 balances TH1/TH17/Treg profile in vivo

**Fig 5:**
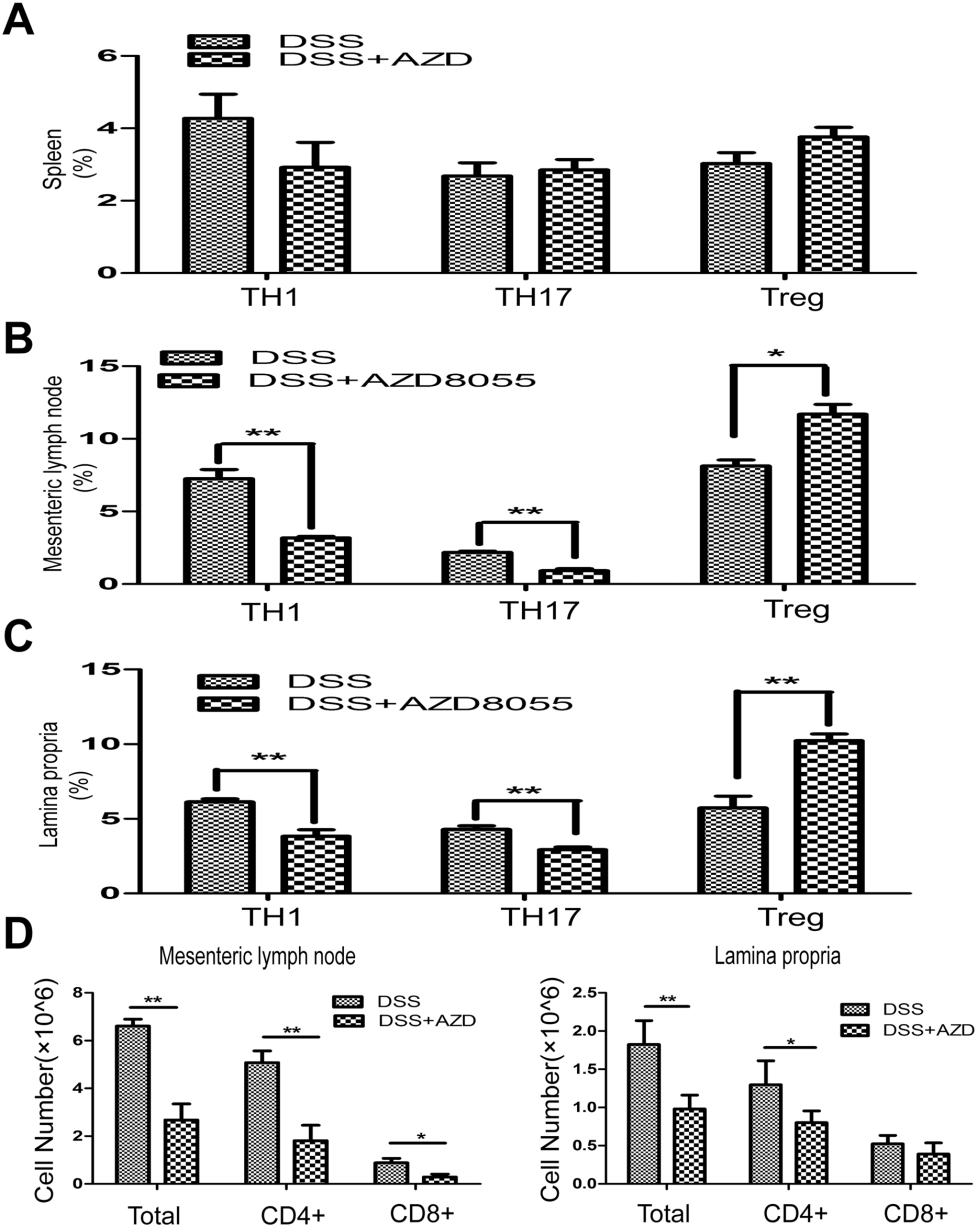
AZD8055 inhibits TH1 and TH17 cell polarization and expands Treg cell polarization in vivo. Lymphocytes were isolated from the spleen, mesenteric lymph nodes and lamina propria and analyzed by flow cytometry on day7. (A) The percentage of TH1 cells (CD4+IFN-γ+), TH17 cells (CD4+IL-17+) and Treg cells (CD4+FOXP3+) in spleen treated with AZD8055 or emulsifier. (B) The percentage of TH1 cells, TH17 cells and Treg cells in mesenteric lymph nodes treated with AZD8055 or emulsifier. (C) The percentage of TH1 cells, TH17 cells and Treg cells in lamina propria treated with AZD8055 or emulsifier. (D) The cell number of CD4+ T cells and CD8+ T cells in the mesenteric lymph nodes and lamina propria treated with AZD8055 or emulsifier. The data were expressed as mean±SEM. Statistical significance was assessed by t-test. *P<0.05, **P<0.01.

Through the activation of separate signaling pathways, naive T cells differentiate into helper T (TH) cells, termed TH1, TH2 and TH17, and induce Treg cells. Dysregulation of TH cells results in IBD. To test the effect of AZD8055 on the proportion of different T helper cell subsets in spleen, mesenteric lymph nodes and lamina propria, we isolated mononuclear cells of mesenteric lymph nodes and lamina propria from DSS-treated mice as well as DSS+AZD8055 treated mice. We found that, although there were no differences in splenic TH1, TH17 and Treg between the control and AZD8055 treated mice (Fig 5A), AZD8055 treatment reduced the proportion of TH1 cells (CD4+IFN-γ+) and TH17 cells (CD4+IL-17+) and increased the proportion of Treg cells (CD4+FOXP3+) in mesenteric lymph nodes and lamina propria (Fig 5B and 5C). In addition, the cell number of CD4+ T cells and CD8+ T cells in the presence of AZD8055 were also decreased in the mesenteric lymph nodes and lamina propria (Fig 5D). Therefore, AZD8055 ameliorated colitis might through balancing TH1/TH17/Treg cell profile to maintain normal immune homeostasis.

### 6. AZD8055 inhibits the proliferation and differentiation of TH1 and TH17 cells in vitro

**Fig 6:**
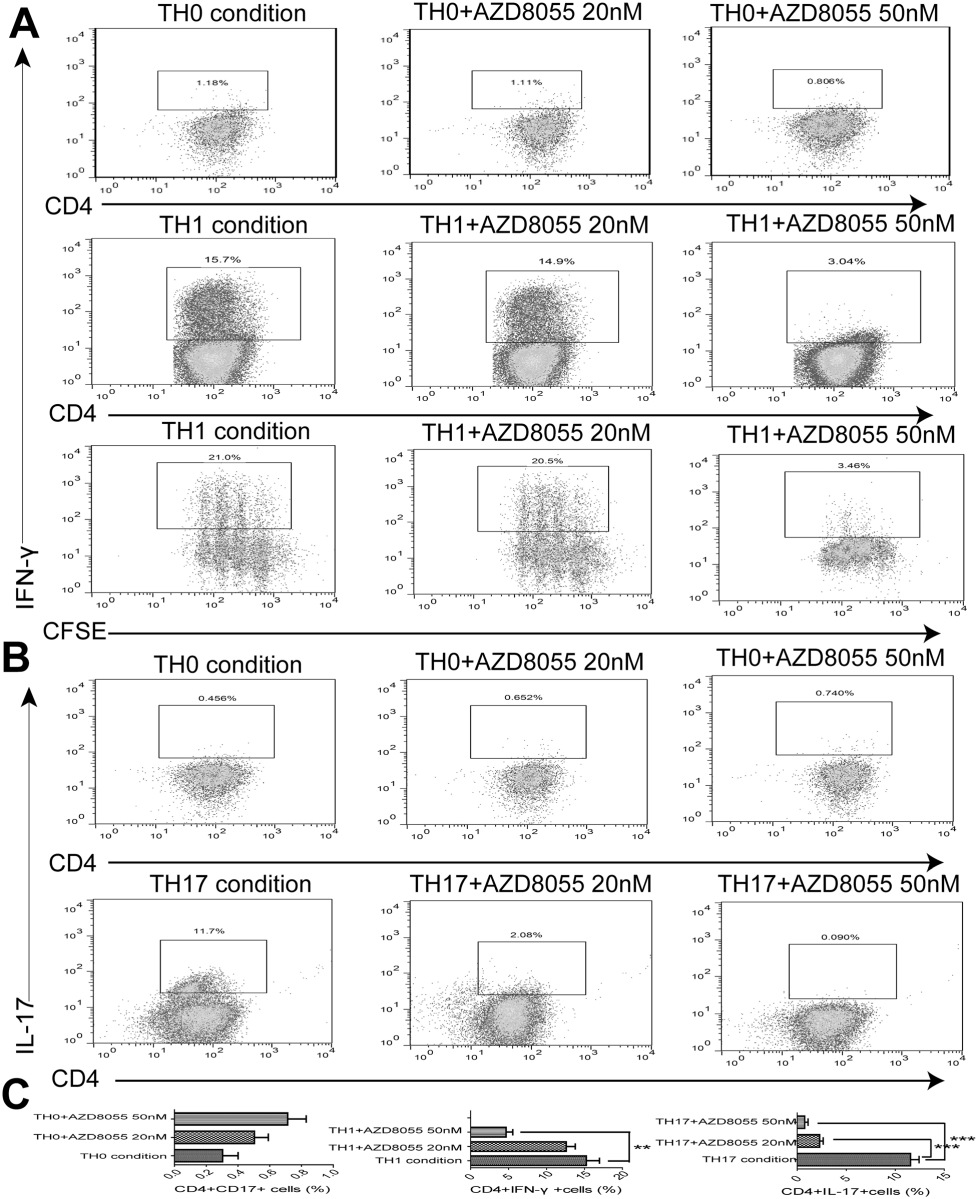
AZD8055 inhibits the proliferation and differentiation of naive CD4+ T cells in vitro. Naive CD4^+^ T cells were isolated from spleen and lymph nodes of mice, CFSE-labeled, and activated in the presence of AZD8055 (0nM, 20nM, 50nM) under TH0, TH1 and TH17 conditions for 4 days. (A) TH1 cells were activated with PMA + Ionomycin and stained for CD4 and intracellular expression of IFN-γ in the presence of AZD8055. Dot-plots showed the expression of CD4+ IFN-γ+ TH1 cells under TH0 condition (Upper panels). Dot-plots showed the differentiation of TH1 cells under TH1 condition (Middle panels). Scatterplot displayed the proliferation of TH1 cells under TH1 condition (Lower panels). (B) TH17 cells were stimulated with PMA + Ionomycin and stained for CD4 and intracellular expression of IL-17 in the presence of AZD8055. Dot-plots showed the expression of CD4+ IL-17 + TH17 cells under TH0 condition (Upper panels). Scatterplot displayed the differentiation of TH17 cells under TH17 condition (Lower panels). (C) Results were from three independent experiments. All results showed the mean±SEM. Statistical significance was determined by student’s t-test. *P<0.05, **P<0.01, ***P<0.005.

Next, we determined if AZD8055 could restrain T cell expansion and differentiation in vitro. CFSE-labeled naive CD4^+^ T cells were isolated and irritated under TH0, TH1 and TH17 conditions for 4 days in the presence of AZD8055 at different concentration (0nM, 20nM, 50nM). We observed a dose-dependent inhibition of TH1 proliferation and differentiation by AZD8055. While there was a significant inhibitory effect of AZD8055 on TH1 cell differentiation at a concentration of 50nM (Fig 6A and 6C). And AZD8055 exhibited the inhibition on TH17 cell differentiation at a concentration of 20nM (Fig 6B and 6C).

### 7. AZD8055 enhances the proliferation and differentiation of Treg cells in vitro

**Fig 7:**
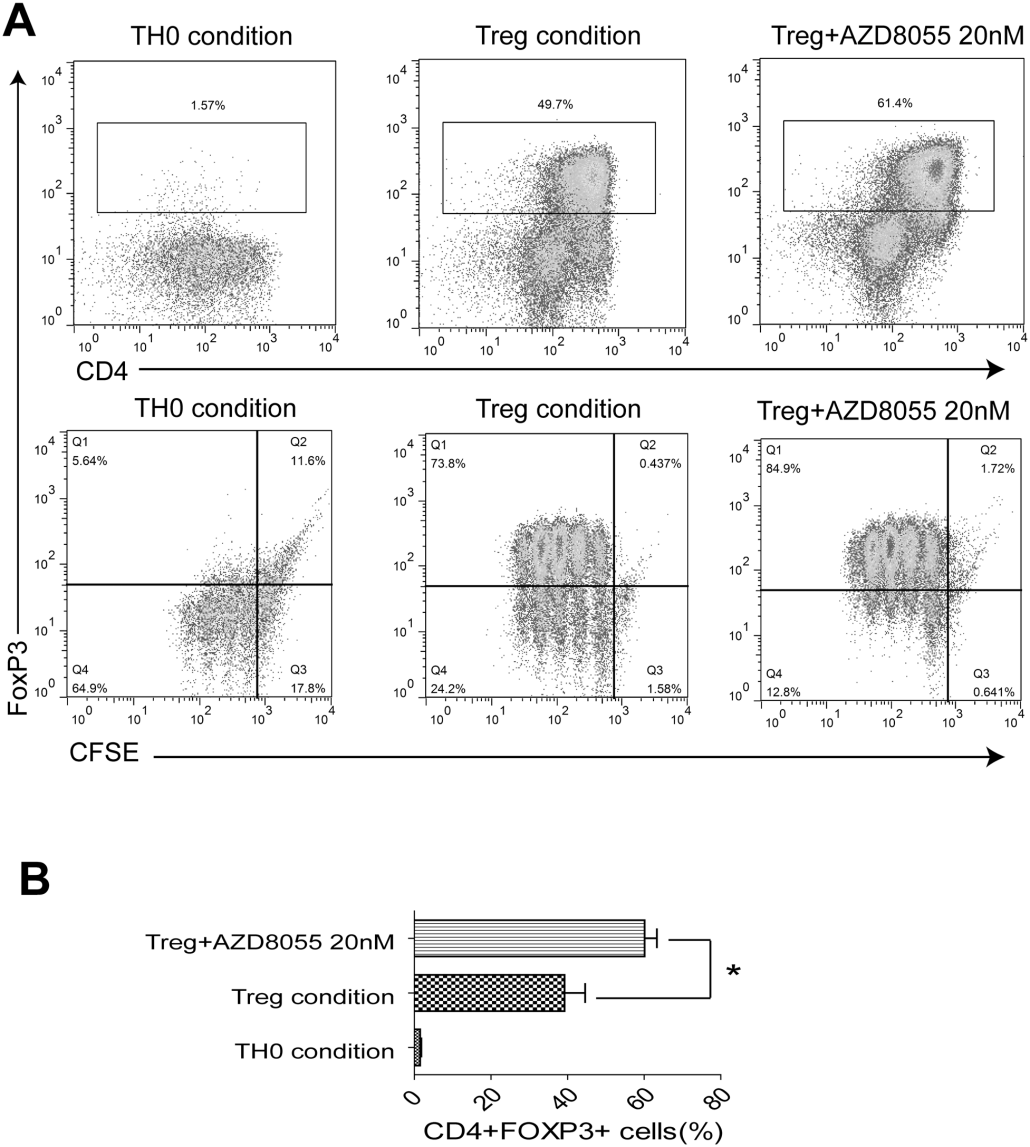
AZD8055 expands the proliferation and differentiation of Treg cells in vitro. Naive CD4^+^ T cells were isolated from spleen and lymph nodes of mice, CFSE-labeled, and activated in the presence of AZD8055 (0nM, 20nM) under TH0 and Treg conditions for 4 days. (A) Treg cells were permeabilized and stained for CD4 and intracellular expression of Foxp3 in the presence of AZD8055 (0nM, 20nM). Dot plots showed the differentiation of CD4+ Foxp3+ Treg cells under TH0 and Treg conditions (Upper panels). Scatterplot displayed the proliferation of Treg cells under TH0 and Treg conditions treated with AZD8055 (Lower panels). (B) Results were from three independent experiments. All results showed the mean±SEM. Statistical significance was evaluated by student’s t-test. *P<0.05, **P<0.01, ***P<0.005.

Then, we evaluated whether AZD8055 could expand Treg cells from CD4^+^ T cells in vitro. CFSE-labeled naive CD4^+^ T cells were cultured in the presence of AZD8055 (0nM, 20nM) under Treg culture condition for 4 days. As shown in Fig 7, the population of CD4+Foxp3+ Treg cells was increased in the presence of AZD8055 at a concentration of 20nM, which might resulted in switching naive T cells differentiation into Treg cells. Consistently, AZD8055 also increased the proliferation of Treg cells at a concentration of 20nM.

## Discussion

In this study, we found that ATP-competitive dual mTOR inhibitor AZD8055 ameliorated DSS-induced colitis by increasing the functional activity of Treg cells and suppressing TH1 and TH17 cell response in vivo. Previous studies have showed that infusion SCID mice with naive T cells without Treg cells induces hyperactivity to intestinal commensal bacteria, while infusing whole T cells prevents inflammation [34–36]. Another study also observed that the depletion of Treg cells in mice deteriorated the disease [37]. In the presence of Interleukin-23(IL-23) or Interleukin-6 (IL-6), naive T cells differentiate into TH17 cells [38, 39]. The efficacy of balancing Treg/TH17 cells has an important role in the clinical and experiment model [40–42]. Tocilizumab, a humanized monoclonal antibody against the membrane and the soluble IL-6, and Ustekinumab, a human monoclonal antibody that blocks IL-12 and IL-23, have been used to treat IBD patients [43, 44]. Hui Yin et al have found that sirolimus (Rapamycin analogue) ameliorated TNBS-induced colitis by promoting differentiation of Treg cells and inhibiting the generation of TH17 cells [45]. In our study, we found that AZD8055 had the dose-dependent effect on inhibition of TH17 cells differentiation, and increase of Treg cells proliferation and differentiation in vitro. Furthermore, we also found that AZD8055 treatment induced the increase of Treg cells and decrease of TH17 cells in the lamina propria and mesenteric lymph nodes in mice. Our results suggested that AZD8055 was involved in the regulation of balancing of Treg/TH17 cells both in vivo and in vitro.

Delgoffe et al reported that mTOR-deficient T cells were unable to differentiate into TH1, TH2, or TH17 effector cells while they displayed normal activation and IL-2 production upon initial stimulation [20]. The incapacity of differentiation was related to STAT activation reducing as well as failure of the up-regulation of lineage specific transcription factors. However, under normal conditions of activation, T cells lacking mTOR were capable of differentiating into Foxp3+regulatory T cells. The results indicated that mTOR pathway was critically involved in the differentiation of CD4+ effector T cells [46, 47]. The first-generation mTOR inhibitors Rapamycin and its analogues such as Sirolimus and Everolimus employed an allosteric mechanism to block mTORC1 output. Meanwhile, second generation mTOR inhibitors such as AZD8055 and PP242 competitively targeted the ATP binding site to hinder kinase activity of both TORC1 and TORC2 [48]. In our study, we found that AZD8055 could suppress the differentiation and proliferation of TH1 and TH17 cells and increase Treg cells both in vivo and in vitro.

Recent studies have found that specific B cell subsets, regulatory B cells (Bregs), are potent immune response regulators and are of crucial importance in a variety of mouse models of immune-mediated disorders like IBD, systemic lupus erythematous(SLE) and rheumatoid arthritis (RA) and immune thrombocytopenia patients(ITP) [49–53]. Flores-Borja et al. investigated that healthy Breg cells especially CD19+CD24hiCD38hi B cells could limit the differentiation of naïve T cells into TH1 and TH17 cells and convert effector CD4+ T cells into Treg cells by producing a large amount of IL-10 [54]. Ashour HM et al. studied that expansion of Breg cells were necessary for the induction of T cell tolerance elicited through the anterior chamber of the eye [55]. Therefore, Bregs may play an important role in the induction of T cell tolerance and in maintaining the key balance between TH1/TH17/Treg populations [56]. In addition, it has also been shown that Granulocyte macrophage colony stimulating factor (GM-CSF) can differentiates precursor cells into tolerogenic DCs that can expand Treg cell numbers and function [57,58]. These Treg cells can suppress effector T cells through secretion of IL-10 [59]. GM-CSF has a critical role in regulating the immune response and maintaining immunological tolerance by inducing specialized cell types from precursors or by affecting phenotypes of mature cell populations [60–62]. The protective effect of GM-CSF has been investigated in IBD and Type 1 diabetes (T1D) [63–66]. Adoptive transfer of GM-CSF could directly expand Treg cells which was shown in diabetes in mouse models [66, 67]. Thus GM-CSF may exert a far-reaching influence on the state of immune tolerance referring to a wide array of autoimmune diseases.

It has been shown that Rapamycin inhibites induced chronic colitis by decreasing leukocyte migration. However, the effect of Rapamycin on T cells regulation has not been investigated. Since Rapamycin also inhibits mTORC2 activity after the prolonged treatment, it would be interesting to compare the efficiency of Rapamycin with AZD8055 in DSS-induced colitis. In our study, we showed that ATP-competitive dual mTOR inhibitor AZD8055 exhibited potent immunosuppressive properties and was used therapeutically in countering autoimmunity and preventing allograft rejection. In future studies, we will use mTORC1 or mTORC2 knockout mice to study the effect of mTOR complexes on DSS-induced mice. We will also investigate the effect of mTOR inhibition on the immune cells from human biopsy in IBD patients and explore possible mechanism.

Although AZD8055 has been used for its anti-tumor activity, its application in the autoimmune diseases still lacks comprehensive understanding [68–71]. The immune regulation of AZD8055 is involved in a variety of immune cells and cytokines. Our study showed that AZD8055 attenuated DSS-induced colitis and also explored its possible mechanisms in immune regulation. This study indicates that ATP-competitive mTOR inhibitor may offer a promising alternative pharmaceutical strategy to manage IBD.

## Abbreviations

ATP: Adenosine triphosphate
mTOR: mammalian target of Rapamycin
DSS: dextran sulfate sodium
RAPA: Rapamycin
IFN-γ: Interferon gamma
TNF-α: tumor necrosis factor alpha
IL-1β: Interleukin-1 beta
TH cell: Helper T cell
TH1 cell: T helper 1 cell
TH2 cell: T helper 2 cell
TH17 cell: T helper 17 cell
Treg cell: Regulatory T cell
Breg cell: Regulatory B cell
GM-CSF: Granulocyte macrophage colony stimulating factor
IBD: Inflammatory bowel disease
CD: Crohn’s colitis
UC: Ulcerative colitis
DMSO: Dimethyl sulfoxide
CFSE: carboxyfluorescein succinimidyl ester
HPRT: hypoxanthine-guanine phosphoribosyl transferase
DAI: disease activity index score
PBS: phosphate buffered saline
PMA: phorbol myristate acetate
