# Correction: mTOR Inhibition Attenuates Dextran Sulfate Sodium-Induced Colitis by Suppressing T Cell Proliferation and Balancing TH1/TH17/Treg Profile

**DOI:** 10.1371/journal.pone.0159758

**Published:** 2016-07-14

**Authors:** Shurong Hu, Mengmeng Cheng, Yilin Wang, Zhengting Wang, Yaofei Pei, Rong Fan

The second author’s name is spelled incorrectly. The correct name is: Mengmeng Cheng. The correct citation is: Hu S, Cheng M, Wang Y, Wang Z, Pei Y, Fan R, et al. (2016) mTOR Inhibition Attenuates Dextran Sulfate Sodium-Induced Colitis by Suppressing T Cell Proliferation and Balancing TH1/TH17/Treg Profile. PLoS ONE 11(4): e0154564. doi:10.1371/journal.pone.0154564
